# Dental Tumor of Jaw

**Published:** 1882-09

**Authors:** 


### DENTAL TUMOR OF JAW.

In the *Canada Medical Record* Dr. C. E. Nelson relates the case of a girl of fifteen, who was suffering from a large tumor of the lower jaw, which had been steadily growing for about six months. For four months the pain had been so severe that morphia had to be used continuously. A distinguished surgeon diagnosed malignant disease, and advised immediate removal of the tumor, along with a considerable portion of the jaw bone. The day before the proposed operation, a dentist was consulted, who, after a careful examination, came to the conclusion that the tumor was not malignant, but was caused by the presence in the jaw bone of several of the second teeth, which had not yet been evolved. The tumor was freely opened, a quantity of extremely fetid matter was discharged, and the pain was instantly relieved. A probe revealed that the anterior surface of the bone below the incisors had been absorbed, and at the bottom of the cavity so formed several hard bodies could be felt imbedded in the bone. The cavity was cleaned out and packed with lint soaked in a solution of chloride of zinc. Anti-septic applications were used regularly, but the wound continued to discharge a thick, black and very fetid fluid. By the end of six weeks three teeth could be distinguished, viz., the right canine and two right lateral incisors, and at the end of three months these teeth were extracted through the opening made when the tumor was lanced. A year afterwards the cavity had filled up and the jaw returned to its normal shape.

